# Annotations

**Published:** 1889-12-14

**Authors:** 


### Annotations.

**Epidemic Influenza.** The influenza which is now prevailing at St. Petersburg seems to be of the true "epidemic" variety. It is, happily, a comparatively unfamiliar affection in this country. There is, however, more than a possibility that we shall shortly have some experience of it. Scientific opinion classes it with hay fever, and it certainly seems to depend on atmospheric conditions of a very peculiar kind, and which travel from east to west. Except to very weakly persons, it is not dangerous to life. But though comparatively little to be feared in that respect, it is an exceedingly distressing and debilitating disease. For three or four days the person attacked continues in a very weak and nervous state, and the effects of the attack do not usually pass away in less than a fortnight. It is, perhaps, more generally and rapidly infectious than any other disease known, and especially among adults. A whole household, master, mistress, and servants, are not infrequently attacked on the same day, and almost at the same hour. Children, strange to say, not seldom escape. Is there any method of prevention? None that we know of that is really effective; but as the infection seems to be in a particular belt of air, if doors and windows could be closed as that belt is approaching, and kept closed until it has passed over, the possibility is that the number of persons attacked might be very much diminished. The belt, as we have said, travels from east to west. When we find that the French people are beginning to be attacked, then is the time to try the experiment of closed doors and windows. We do not say this precaution will be effectual to prevent, but it is well worth a trial. Possibly respirators to cover both mouth and nose might also be used with advantage. The instrument makers ought to have some in readiness. Forewarned should at least try to be forearmed. Perhaps, after all, there is more alarm than the occasion warrants. But it is always well to try reasonable experiments when there is no known successful method of treatment.

**The Parish Doctor.** It seems a hard case when a parish doctor refuses to attend a patient if called, merely on the ground that he has not received an "order" from the proper quarter. If the patient suffers much, or, still worse, dies before the usual formalities are complied with, everybody is ready at once to cry out about the hard-hearted selfishness of the doctor. We do not recognise it as any part of our duty to defend the medical or any other profession "through thick and thin," but we do desire to see bare justice done, even to doctors. A case has just been decided before Mr. Justice Cave and a special jury which goes to show that in certain instances of seeming hardship the parish doctor may be absolutely in the right, and the patient's friends as unquestionably in the wrong. It appears that Mr. Inglis Mason, surgeon and medical officer for Sudbury, refused to attend the sick child of a woman named Sharman unless she either obtained an "order" from the relieving officer, or paid a stipulated fee. The child died, and a local newspaper thereupon described the doctor's conduct as parsimonious and heartless, and also reflected upon Mr. Mason in connection with some other cases. The jury, after the summing up of the judge, and without retiring from the box, gave a verdict for the doctor with costs. It is plain that in this instance the evidence was such as to show at once and conclusively that the medical man had committed no fault. It consists with our experience that in at least nineteen-twentieths of similar cases it is the parents or friends who are at fault, and not the doctor. As a rule the parish medical officer does at least three times as much for the poor as he is paid for, and does it to the best of his ability. It is proved beyond question that the medical profession is most humane, and that the members of it are ready to run here and there at a moment's notice without thinking of fee or reward. If, however, the doctor were always to go without an "order" whenever he is called, it is certain that no trouble would ever be taken to procure proper "orders," and that his calls would be so numerous that his life would be worse than that of a slave or a dog. Nobody is so inconsiderate with medical men as the poor; and of the poor the actual paupers are by far the worst. When a doctor of established character is charged with neglect of duty, the public will do well in all cases to suspend judgment until the charge is proved beyond dispute.

**The Judge and the Professor.** In this country the shining lights of one profession do not always attach very much importance to the opinions of the distinguished luminaries of another. Doctors of divinity have been known to speak very slightingly of doctors of physic, and, it must be admitted, that doctors of physic, though of course very rarely, have returned the compliment. That is exactly as it should *not* be. The man who has tasted of the finest fruits of culture in one field always values very highly the attainments and judgments of his fellow-man who has been similarly privileged in another. The specialist in one department of intellectual activity who thinks slightingly of the specialist in another department, thereby proves, not only that he is a person of inferior culture, but, what is much worse, of ignoble temper. This preface is by way of bespeaking at the hands of medical and other examiners a careful consideration of the opinions expressed by two very eminent persons the other day on the subject of examinations in general. Those two persons were Lord Coleridge and Prof. Henry Morley. Mr. Morley, speaking to old and present students of the Stockwell-road Training College, said that in "the present system of education there was a great deal too much grinding, and a great deal too much examination." Lord Coleridge, when it came to his turn to speak, said he thanked Mr. Morley for his "denunciation of examinations." He often put up a "silent prayer of thanks for the fact that he was educated in the pre-examination period." Those who are responsible for the present system of examination should give to these, evidently deeply-pondered opinions, the thought and consideration they certainly deserve. It is said that the examination system, as at present conducted, has been long enough on its trial to show what it is really worth. One definite result is claimed for it—viz., that it "has filled almost every academic post in the kingdom with 'pedants' and left the 'brains' to circulate in the world at large." It must be confessed that there is a very great deal of truth in this. The non-examined Milton wrote "Paradise Lost," and the non-examined Tennyson "The Idylls of the King." The non-examined Shakespeare wrote "Hamlet" and "Macbeth," and the non-examined Goethe gave us "Faust." Augustine belonged to the pre-examination period, and so did Bishop Butler. To that period, too, belonged Locke and Macaulay, Bacon and Newton, Harvey and Sydenham, Hunter and Bell. Where are their successors among the much-examined men of to-day? We do not object to examinations if they test a man's brains and thinking capacity. But when they merely test, as they do at the present time, his power of sustained "cram," they are about as wise and as useful as the stuffing of a miserable dyspeptic with Christmas puddings, Scotch buns, and new port wine. Nothing is either so valuable or so rare as original thinking power, and its rarity was never more conspicuous than it is to-day in every academic, literary, and medical circle of our much-examined country.

